# Transcriptomic and Immunohistochemical Profiling of SLC6A14 in Pancreatic Ductal Adenocarcinoma

**DOI:** 10.1155/2015/593572

**Published:** 2015-05-27

**Authors:** Alan R. Penheiter, Sibel Erdogan, Stephen J. Murphy, Steven N. Hart, Joema Felipe Lima, Fariborz Rakhshan Rohakhtar, Daniel R. O'Brien, William R. Bamlet, Ryan E. Wuertz, Thomas C. Smyrk, Fergus J. Couch, George Vasmatzis, Claire E. Bender, Stephanie K. Carlson

**Affiliations:** ^1^Department of Molecular Medicine, Mayo Clinic, 200 First Street SW, Rochester, MN 55905, USA; ^2^Department of Biochemistry and Molecular Biology, Mayo Clinic, 200 First Street SW, Rochester, MN 55905, USA; ^3^Biomedical Statistics and Informatics, Mayo Clinic, 200 First Street SW, Rochester, MN 55905, USA; ^4^Medical Genome Facility, Mayo Clinic, 200 First Street SW, Rochester, MN 55905, USA; ^5^Department of Epidemiology, Mayo Clinic, 200 First Street SW, Rochester, MN 55905, USA; ^6^Department of Anatomic Pathology, Mayo Clinic, 200 First Street SW, Rochester, MN 55905, USA; ^7^Department of Laboratory Medicine and Pathology, Mayo Clinic, 200 First Street SW, Rochester, MN 55905, USA; ^8^Department of Radiology, Mayo Clinic, 200 First Street SW, Rochester, MN 55905, USA

## Abstract

We used a target-centric strategy to identify transporter proteins upregulated in pancreatic ductal adenocarcinoma (PDAC) as potential targets for a functional imaging probe to complement existing anatomical imaging approaches. We performed transcriptomic profiling (microarray and RNASeq) on histologically confirmed primary PDAC tumors and normal pancreas tissue from 33 patients, including five patients whose tumors were not visible on computed tomography. Target expression was confirmed with immunohistochemistry on tissue microarrays from 94 PDAC patients. The best imaging target identified was SLC6A14 (a neutral and basic amino acid transporter). SLC6A14 was overexpressed at the transcriptional level in all patients and expressed at the protein level in 95% of PDAC tumors. Very little is known about the role of SLC6A14 in PDAC and our results demonstrate that this target merits further investigation as a candidate transporter for functional imaging of PDAC.

## 1. Introduction

Early detection and surgical resection of pancreatic ductal adenocarcinoma (PDAC) confined to the pancreas offers the best hope for cure or extension of lifespan. Recent breakthroughs in serum profiling, most notably mass spectral and antibody array technologies, provide hope for screening patients with asymptomatic disease [1, 2]. However, even with screening, two critical problems remain: (1) localization of small or diffusely infiltrative occult lesions in the pancreas and (2) detection of small metastatic deposits.

The majority of large PDACs are detected with anatomical imaging techniques such as computed tomography (CT), magnetic resonance imaging (MRI), and ultrasound. Multidetector, helical CT with intravenous administration of contrast material is the most commonly used imaging procedure to detect and stage suspected PDAC. Diagnostic accuracy decreases, however, with decreasing tumor size [3–5] and in patients with chronic pancreatitis [6, 7]. There is also a subgroup of tumors (about 10%) that are isoattenuating to normal pancreas. These are typically diffusely infiltrative rather than mass-forming, which renders them invisible on CT despite tumor dimensions greater than the expected size for detection [8, 9]. Functional imaging with 2-deoxy-2-[^18^F]fluoro-D-glucose positron emission tomography (^18^FDG-PET), combined with CT or MRI, is a highly sensitive diagnostic tool for many tumor types, but its utility in PDAC is hampered by low tumor signal-to-background ratios that limit its sensitivity for detection of lesions below the realized resolution of PET (approximately 1 cm). A new functional imaging probe that selectively targets PDAC with high sensitivity is a critical unmet need in PET/CT or PET/MRI that could transform patient management by allowing earlier PDAC detection and surgical intervention and that could improve preoperative staging of disease to decrease the number of unwarranted surgeries in patients who might benefit from experimental systemic therapy.

One of the greatest success stories in functional imaging is sodium iodide symporter- (NIS-) mediated imaging for thyroid cancer. NIS is a membrane transporter from the SLC family (SLC5A5) that is responsible for the uptake of iodine in thyroid follicular cells as the first step in the synthesis of thyroid hormone. The combined action of NIS and a second trapping (or organification) step allow thyroid cancer cells to accumulate radiolabeled iodine >1000-fold above blood levels at 48 hours after administration [10]. This efficient 2-compartment system permits highly sensitive detection of primary and metastatic thyroid cancer deposits with gamma-camera, single-photon emission computed tomography (SPECT), and PET imaging as well as the effective use of ^131^I (iodine 131) radiotherapy for thyroid cancer. Another well-characterized SLC family member useful in functional imaging is SLC2A1 (GLUT1), a major glucose membrane transporter that is upregulated in tumor cells (glycolytic rates in tumors can be more than 30-fold higher than in normal cells) [11, 12]. ^18^FDG is a glucose analog that is taken up by GLUT 1 in tumor cells and phosphorylated by hexokinase, thus trapping it in the cell. This accumulation of ^18^FDG within tumor cells serves as the basis for PET imaging of a wide variety of cancers.

Our previous work with NIS [13–15] and the success of SLC transporter-mediated functional imaging in other tumor models led us to investigate the potential of SLC-mediated functional imaging for PDAC. In this study, we performed gene expression profiling in human PDAC samples using laser capture microdissection (LCM) and RNA sequencing (RNASeq) to search for upregulated SLC transporters in PDAC compared with normal pancreatic tissue and normal liver tissue (the major site of PDAC metastases). Our transcriptomic results were validated at the protein expression level using immunohistochemistry and tissue microarray analysis. The top candidate transporter (upregulated at the transcriptional level in all patients studied) identified in our search was SLC6A14.

SLC6A14 (also known as ATB0, +) is a Na+ and Cl− dependent solute transport system in the SLC6 family that is capable of active transport of all neutral and basic amino acids except glutamate and aspartate which are nonessential [16, 17]. Because of its broad substrate specificity and the fact that the transporter is expressed only at low levels in many normal tissues, SLC6A14 has received considerable attention for its potential as a drug delivery system. Recent studies have shown the anticancer potential of inhibitors of this transporter system [12, 18–21]. SLC6A14 has been shown to be upregulated in primary and metastatic colorectal cancer [21], cervical cancer [22], and estrogen receptor-positive breast cancer [18]. Kandaswamy et al. recently determined that SLC6A14 is upregulated severalfold in cultured pancreatic cancer cell lines (compared with the normal ductal cell line) and that blockade of the transporter with *α*-methyltryptophan led to decreased proliferation of PDAC cells in vitro and in a mouse xenograft model [23]. However, very little is known about SLC6A14 expression, subcellular localization, and transporter function at the protein level in any of these tumor types. The goal of our study was to further investigate the expression of SLC6A14 in primary human PDAC tissues and determine its potential as a target for the development of a new functional diagnostic imaging probe.

## 2. Materials and Methods

In addition to our retrospective review of patients' medical records, we analyzed PDAC specimens contained in the institutional pancreatic cancer tissue registry after approval of our study protocol by the Mayo Clinic Institutional Review Board. Between January 1, 2002, and September 30, 2012, a total of 616 patients with surgically resected PDAC had consented to be included in the pancreatic cancer tissue registry, which is maintained by the Mayo Clinic SPORE (Specialized Program of Research Excellence (National Cancer Institute)) in Pancreatic Cancer. Tissues from a total of 33 patients were included in our transcriptomic studies, and tissues from a total of 94 patients were included in our protein expression-profiling studies.

### 2.1. Microarray Analysis

We first performed microarray analysis on frozen bulk PDAC tumor samples from 12 patients and 5 (matched) normal human pancreas samples. Tissues were mounted on glass slides and macrodissected with razor blades. RNA was isolated and characterized as described below with the LCM-captured samples. Complementary DNA (cDNA) libraries constructed from the purified RNA were run on a U133 plus 2.0 Affymetrix (Santa Clara, CA) array.

### 2.2. Laser Capture Microdissection (LCM)

LCM was performed on frozen pancreatic tissues from an additional 21 patients including 5 with CT-isoattenuating tumors (described below) from the pancreatic cancer tissue registry with histologically confirmed PDAC for whom both tumor and normal pancreas tissues were available. Tissues were cut into 10 *μ*m sections on nuclease-free polyethylene naphthalate (PEN) membrane slides (Carl Zeiss, Thornword, NY) prechilled at 4°C. After tissues were sectioned, the slides were placed on dry ice and then stored at −80°C. Slides to be microdissected were removed from freezer storage, stained with cresyl violet in accordance with the Ambion LCM Staining Kit protocol (Life Technologies, Grand Island, NY), dehydrated with two 90-second xylene washes, and allowed to air-dry for 5 minutes. Regions of interest from the dehydrated tissue sections were subsequently microdissected from the PEN membrane slides using an Arcturus Veritas Laser Capture Microdissection instrument (Life Technologies), utilizing both its ultraviolet cutting laser and its infrared capture lasers. Captured cells were collected onto CapSure Macro LCM caps (Life Technologies). The amount of tissue on each cap was approximately one-third to one-half the area of the LCM cap. Multiple LCM caps were used, as needed, for each patient case. After microdissection, each LCM cap was immediately placed on top of a 0.5 mL polymerase chain reaction (PCR) tube containing 125 *μ*L of QIAzol lysis reagent (QIAGEN, Germantown, MD), which was then inverted to allow the reagent to cover the captured tissue before it was placed on dry ice. These samples were then stored at −80°C until processed for RNA extraction.

### 2.3. Patient and Imaging Data Collection for CT-Isoattenuating Tumors

To ensure that our results were applicable to CT-isoattenuating tumors and that these tumors were similar at the transcriptional level to the classic hypoattenuating PDAC tumors, we searched the pancreatic cancer tissue registry to identify a subset of CT-isoattenuating cases. We searched for patients who had histological confirmation of pancreatic adenocarcinoma on resected tissue but did not have a mass evident on a preoperative contrast-enhanced CT scan. Of the 616 patients in the registry, 49 had both frozen tumor and frozen normal pancreatic tissue as well as a CT scan prior to treatment which were available for review.

All 49 patients had undergone high-resolution, thin-section, dynamic, intravenous contrast-enhanced imaging on multidetector row CT scanners using a pancreatic protocol that included biphasic imaging in the pancreatic phase (approximately 45 seconds after contrast injection) and in the portal venous phase (70 seconds after contrast injection). Initial review of the CT images by an abdominal radiologist (S. K. C.) on our institution's imaging archive software identified 5 patients (3 men, 2 women; mean age, 59 years (range, 47 to 76 years)) with pancreatic tumors that appeared to be CT-isoattenuating (imperceptible changes in contrast to the adjacent pancreatic parenchyma upon visual inspection in both the pancreatic and portal venous phases of contrast enhancement), so they were included in this study.

### 2.4. RNA Isolation

RNA was extracted from all dissected tissues using the miRNeasy Mini Kit protocol and the automated QIAcube instrument (QIAGEN). For cases in which multiple LCM caps were used to collect an identical cell type, the extracted material from those LCM caps was consolidated.

### 2.5. mRNASeq Library Preparation and Sequencing of Formalin-Fixed Paraffin-Embedded-Derived RNA

RNA libraries were prepared according to the manufacturer's instructions for the NuGEN Ovation RNASeq FFPE (formalin-fixed paraffin-embedded) and Ultralow Library Systems (San Carlos, CA). Briefly, first strand cDNA was generated from 10 ng of total RNA using DNA/RNA chimeric primers and reverse transcriptase to create a cDNA/RNA hybrid. Second strand cDNA was then synthesized containing a DNA/RNA duplex. The resulting double-stranded cDNA molecule was amplified by Single Primer Isothermal Amplification (SPIA), using a chimeric SPIA primer, DNA polymerase, and RNase H (NuGEN). After amplification, the products were modified by random priming and extension to create double-stranded products suitable for generating libraries for sequencing. The double-stranded products underwent blunt-end repair, and unique index molecules were ligated to the 5′ and 3′ ends of each fragment to facilitate PCR amplification of the fragments and to produce the final library. The concentration and size distribution of the resulting libraries were determined on an Agilent Bioanalyzer DNA 1000 chip (Santa Clara, CA) and then confirmed by Qubit fluorometry (Life Technologies). Libraries were loaded onto paired-end flow cells with 3 samples per lane at concentrations of 8 to 10 pM to generate cluster densities of 700,000/mm^2^ following the standard protocol using the Illumina cBot and cBot paired-end cluster kit version 3 (San Diego, CA). The flow cells were sequenced as 51 × 2 paired-end reads on an Illumina HiSeq 2000 using TruSeq SBS sequencing kit version 3 and SCS version 1.4.8 data collection software. Base-calling was performed using Illumina's RTA version 1.12.4.2.

### 2.6. RNASeq Data Acquisition and Analysis

The Mayo Analysis Pipeline for RNA Sequencing (MAP-RSeq), developed by the Bioinformatics Core at Mayo Clinic, was used to perform the thorough processing of the paired-end RNASeq data. MAP-RSeq integrates a suite of open source bioinformatics tools with in-house developed methods to analyze paired-end RNASeq data. The application processes the reads produced by the sequencer in the following manner: quality control, genomic alignments, reference and novel transcriptomic junction alignments, alignment cleanup, identification of genomic features per sample, and summary of data across samples. Postanalysis quality control measures were taken computationally and manually to verify that the analysis was successful. Such computational measures included estimating the distance between paired-end reads, sequencing depths at alternate splicing sites, the rate of duplicate reads, and evaluating coverage across genes. These and other metrics were measured using RSeQC software [24]. Paired-end reads were aligned by TopHat 2.0.6 [25] against the hg19 genome build with the Bowtie1 aligner [26] as a backbone. Gene counts for evaluating RNA fold change within and across samples were generated using HTseq software (http://www.huber.embl.de/users/anders/HTSeq/doc/overview.html) and the gene annotation files used were obtained from Illumina (http://cole-trapnell-lab.github.io/cufflinks/).

### 2.7. Tissue Microarray Screening

An adenocarcinoma tissue microarray (TMA) containing samples from 140 patients without prior chemotherapy was stained with hematoxylin-eosin or colorimetrically developed with antibodies against SLC6A14 or KRT19 (positive control cytokeratin 19) in the Pathology Research Core of Mayo Clinic, Rochester, MN. TMA slides were placed in the BOND III (Leica Biosystems, Chicago, IL, USA) stainer for online processing. Slides were treated with Epitope Retrieval 2 solution for 20 minutes, stained with rabbit polyclonal anti-SLC6A14 (Sigma, St. Louis, MO, HPA003193) at 1 : 100 dilution (in Bkg reducing diluent, Dako, Carpinteria, CA, S3022) for 30 minutes or mouse monoclonal anti-KRT19 (Dako, RCK108) at 1 : 200 for 15 minutes. Detection was achieved using the Polymer Refine Detection kit following the manufacturer's instructions (Leica Biosystems). Counterstaining was performed for 5 minutes with hematoxylin-eosin. Slides were dehydrated through increasing concentrations of alcohol, cleared in xylene, and coverslipped in xylene-based mounting media. The TMAs were evaluated microscopically at 400x magnification for SLC6A14 expression by a trained pancreatic pathologist and were scored as strong, moderate, weak, or absent. Subcellular localization of the staining was noted for each core. In the final analysis, 174 tumor cores from 94 unique patients retained sufficient tissue on the slide with a large enough region of histologically identifiable adenocarcinoma to permit scoring. Information across the multiple evaluable cores per patient was reduced to one observation per unique subject by using the core which stained with the highest expression.

## 3. Results

### 3.1. Transcriptomic Profiling Studies

**Figure 1:**
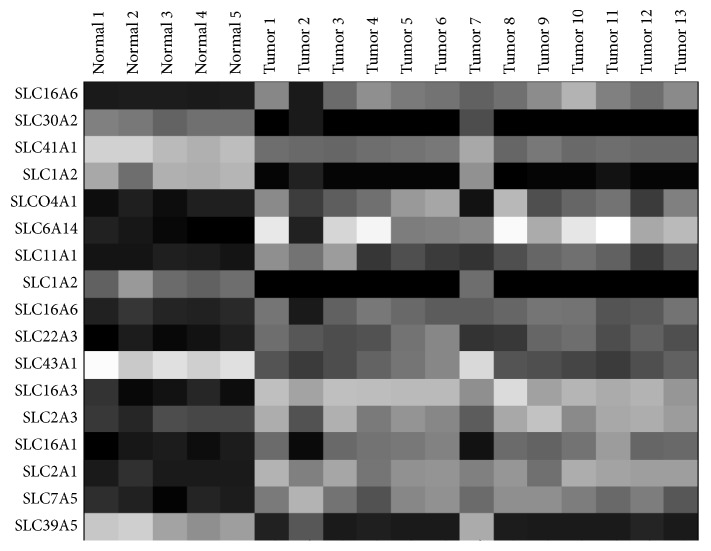
Heat map of transcriptomic analysis. Analysis shows results for pancreatic ductal adenocarcinoma samples from 12 patients (samples 5 and 6 are from 1 patient) and 5 matched bulk normal pancreas patient samples. Samples were macrodissected and run on an Affymetrix U133 plus 2.0 array. Black indicates low level of transcripts and white indicates high level of transcripts. SLC1A2 and SLC6A16 were detected by two probes in the array. All SLC transporters shown were significantly (*P* < 0.05) upregulated or downregulated compared to normal pancreas.

Our work with NIS led us to investigate the differential expression of other metabolic genes in the SLC family that may be upregulated in PDAC cells compared with normal pancreas. We first performed a pilot study of 12 macrodissected frozen human PDAC samples and 5 (matched) normal human pancreas samples. We isolated RNA from these samples and ran libraries on a U133 plus 2.0 Affymetrix array. In these samples, we found 15 SLC transporters that were overexpressed in bulk tumor tissue versus normal tissue (Figure 1). The most promising upregulated candidate gene was the amino acid transporter SLC6A14. This technique also identified the glucose transporter SLC2A1 and the lactate exporter SLC16A3 (Figure 1). SLC2A1 and SLC16A3, along with hexokinase II, facilitate trapping of ^18^FDG. However, PDAC tumors have a large stromal cell component and it was not clear which of the SLCs were specifically upregulated in tumor epithelial cells or which were simply present on expanding or invading stromal cells, such as myofibroblasts, macrophages, and lymphocytes.

**Figure 2:**
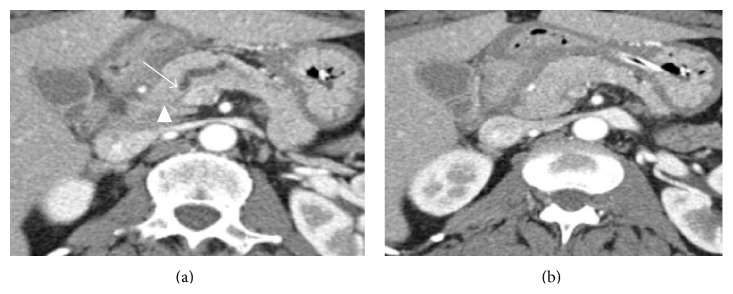
Contrast-enhanced computed tomography images obtained during the pancreatic phase. (a) Axial CT image of the pancreatic head shows abrupt termination of a markedly dilated pancreatic duct (arrow) due to a surgically proven pancreatic cancer causing duct obstruction. No mass is visualized in the region of the duct-obstructing tumor (arrowhead). (b) Image of the pancreatic head 1 cm inferior to image (a) shows homogeneous enhancement without evidence of hypoattenuation changes or mass effect despite the presence of large (3.9 cm) diffusely infiltrating pancreatic ductal adenocarcinoma tumor.

To specifically address the issue of cell-type contribution, we used LCM to isolate PDAC cells and control cells in an additional 21 PDAC patient samples including 5 tumor and matched normal pancreas frozen specimens from patients with CT-isoattenuating tumors that were not visualized on preoperative CT imaging studies (Figure 2). We then purified RNA from the samples and performed RNASeq analysis. As we found in the bulk tumor samples, SLC6A14 was highly overexpressed in this patient group after LCM (Table 1). SLC6A14 was overexpressed at least 2-fold in all 21 patients versus normal pancreas (Table 1). For the comparison with tumor tissue, we harvested 3 different control tissues. Adjacent normal ducts were collected from all 16 patients not selected for CT-isoattenuating characteristics. Acinar tissue adjacent to the tumor was also recovered from the slides of 5 patients. These tumor-adjacent controls provide some insight into whether a gene is upregulated specifically in adenocarcinoma cells or is simply a response of epithelial cells to the tumor microenvironment. For the CT-isoattenuating cases, we used acinar tissue from a region of the resected pancreas distant from the tumor. The distant acinar control was included because it best reflects the background one would encounter when imaging a pancreas from a patient without cancer.

### 3.2. Protein Expression Profiling Studies

**Figure 3:**
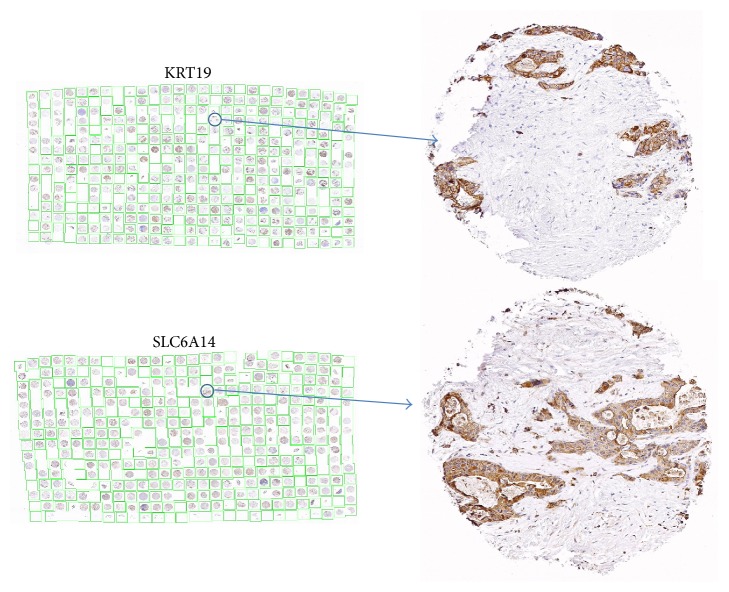
Representative core staining of pancreatic adenocarcinoma by tissue microarray. Top panel was stained with an antibody to KRT19 (a marker for all pancreatic ductal cells). Bottom panel was stained with an antibody to SLC6A14.

Next we sought to determine whether SLC6A14 transcript upregulation resulted in relevant upregulation at the protein level. For this analysis, we used a pancreatic cancer TMA and immunohistochemistry with an antibody against SLC6A14 (Figure 3). A total of 174 tumor cores from 94 patients were evaluated for SLC6A14 staining intensity, percentage of tumor cells stained for SLC6A14, and subcellular localization of SLC6A14 staining. Of the 174 cores, 16 were scored as strong staining, 41 as moderate, 97 as weak, and 20 as negative for SLC6A14. The 154 cores with weak to strong staining were then scored for the percentage of tumor cells within the core that were stained for SLC6A14. Of these, 116 cores had >75% of their tumor cells stained, whereas 16, 14, and 8 cores exhibited staining in 50%–75%, 25%–50%, and <25% of tumor cells, respectively. Staining was predominantly cytoplasmic in 153 cores; distinct plasma membrane staining was found in only 1 core. Of the 153 cores with predominantly cytoplasmic staining, 3 were noted to also have staining of the plasma membrane, and 10 were noted to have additional nuclear staining. Figure 3 illustrates a core that was scored as strong for SLC6A14 along with an adjacent section of the TMA developed for KRT19 (a marker for all pancreatic ductal cells) immunohistochemistry. KRT19 staining was also scored as strong.

**Figure 4:**
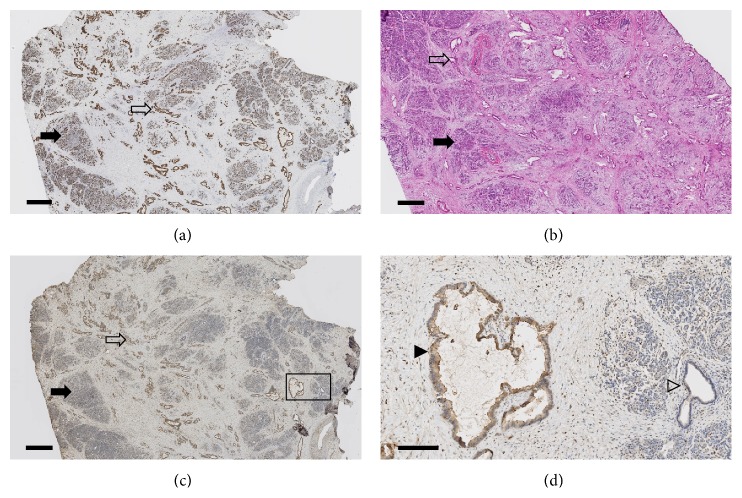
Histology of CT-isoattenuating tumor. (a–c) Note the diffusively infiltrative invasive adenocarcinoma with ductal morphology (open arrows), the relatively intact lobular architecture of the residual normal pancreas (solid arrows), and the regions of fibrosis with sparse cellularity (area between arrows). Scale bar equals 1 mm. (a) KRT19 immunohistochemistry of a formalin-fixed, paraffin-embedded (FFPE) section. (b) Hematoxylin-eosin stain of a frozen section from the same patient. An adjacent section from this same frozen block was used for laser capture microdissection. (c) An adjacent section from the FFPE block shown in panel (a) stained for SLC6A14. (d) Higher magnification of region of panel (c) (rectangle) shows moderate cytoplasmic SLC6A14 staining in adenocarcinoma cells (solid arrowhead), while the adjacent normal acinar cells and a normal duct (open arrowhead) are unstained. Strong cytoplasmic/membranous staining was also observed in scattered stromal cells. Scale bar equals 200 *μ*m.

Whole sections from the 5 CT-isoattenuating cases were also processed for SLC6A14 immunohistochemistry. Four cases were found to have moderate cytoplasmic staining and 1 case was found to have strong cytoplasmic staining. A representative case of moderate cytoplasmic staining is shown in Figure 4. This case shows the diffusely infiltrating pathology that is a hallmark of CT-isoattenuating pancreatic adenocarcinoma histology [9, 27].

No staining of pancreatic epithelial cells (acinar cells or duct cells) or hepatocytes was observed on control tissue cores included on the TMA (3 cores of normal pancreas and 3 cores of normal liver). Occasional moderate to strong staining was observed in other cell types in the control tissues, including stellate cells in the pancreas and Kupffer cells in the liver. Lymphocytes within vessels in the control tissues were also stained for SLC6A14. A complete catalog of normal tissue staining with the SLC6A14 antibody we used can be found on the website of the Human Protein Atlas (http://www.proteinatlas.org/) [28].

We also examined the relationship between SLC6A14 staining intensity and patient demographics, including survival after resection, tumor stage, and tumor grade (Table 2). For this analysis, patients with more than 1 core on the TMA were grouped by the core with the highest intensity of the SLC6A14 staining. Most (95% (89/94)) of the patients had 1 or more cores exhibiting weak or greater SLC6A14 staining. No significant relationship between SLC6A14 staining intensity and patient or histological parameters was found.

## 4. Discussion

Traditional strategies for imaging SLCs (metabolic imaging) have followed a probe-centric paradigm. Radiolabelled “nutrients” were synthesized based on the concept that tumor cells are metabolically active and must upregulate transport and metabolic machinery. Trial-and-error biodistribution studies were used to determine (1) which tumor types were best visualized by a particular probe, (2) whether background uptake might interfere with localization, or (3) whether an unacceptable dose of radiation had been administered to a normal tissue in extreme cases. Some minimal mechanistic studies with inhibitors (primarily at the cell culture level) have been used to identify transporters or transporter families responsible for metabolic imaging probe accumulation, but in many cases the transporters responsible for successful metabolic imaging are not known. This probe-centric approach has been quite successful. Notable achievements are L-[^18^F-fluoro]dihydroxyphenylalanine [29, 30] for detection of neuroendocrine tumors, L-[^11^C-methyl]methionine [31, 32] for many types of extracranial tumors, and ^11^C-choline or ^18^F-choline [33, 34] for detection of primary and metastatic prostate cancer. However, for pancreatic cancer, none of these strategies has been successful enough to reach routine use in clinical practice. The main limitation is the high metabolic and biosynthetic activity of the normal pancreas, which results in massive accumulation of these probes [35–38].

Here we used a target-centric strategy using transcriptomics and protein expression profiling from large numbers of resected patient tissues. The aim was to find a particular transporter that is specifically upregulated in pancreatic cancer versus normal pancreas that could serve as a target for the future development of a PDAC-selective functional imaging probe. Surprisingly, we found that SLC6A14 was overexpressed at the transcriptional level in all the patient samples we studied when these were compared to either microdissected normal ducts or macro- or microdissected acinar tissue. Additionally, at the protein level, SLC6A14 staining of weak or greater intensity was observed in 95% of patient samples, with moderate to strong staining in 41% of patient samples.

We also confirmed that SLC6A14 is overexpressed in CT-isoattenuating PDAC, a subset of tumors with histological and enhancement characteristics that make them notoriously difficult to detect with anatomical imaging techniques. While >90% of PDAC tumors (in symptomatic patients) present at a late stage and are readily detectable by dual-phase contrast-enhanced CT, 5% to 10% of patients present with tumor masses that are not visualized on CT [8, 9, 27]. In most cases, the CT-isoattenuating tumors on gross pathology are much larger than the resolution of CT but are diffusely infiltrating with sparse tumor tissue and abundant normal tissue, and thus they do not appear as a distinct locus of hypoattenuation [9, 27].

For the purposes of functional imaging, a transporter protein needs to be expressed on the plasma membrane in order to facilitate entry of a probe into the target cells. While the function of SLC6A14 is being a plasma membrane transporter, staining was predominantly cytoplasmic in the majority of resected PDAC tissues in our study. A characteristic of this and other related plasma membrane transporters is a predominantly cytoplasmic localization (most likely sequestered in vesicles) with translocation to the plasma membrane after a stimulus [39]. For SLC6A14, protein kinase C alpha activation results in a dramatic translocation to the membrane and subsequent increase in transport activity [39]. At this point, we can only speculate as to why SLC6A14 was predominantly cytoplasmic in our PDAC samples. One possibility is that the loss of blood supply prior to surgical resection may have resulted in the downregulation of signals required for membrane localization of SLC6A14. Interestingly, in other tumor types (thyroid, cervical, and squamous cell carcinoma of the head and neck), strong membrane staining has been observed with the same SLC6A14 antibody (http://www.proteinatlas.org/) [28].

Further understanding of the function, substrate specificities, and tissue distribution of SLC6A14 will aid in the potential future development of a selective functional imaging probe or the discovery of targeted therapeutic agents. An exciting development towards this goal was the recent synthesis of* O*-2((2-[^18^F]fluoroethyl)methylamino)ethyltyrosine or ([^18^F]FEMAET), a cationic amino acid PET probe that demonstrates positive uptake in SLC6A14-positive xenografts [40, 41]. Anticancer strategies (transporter inhibitors to starve tumor cells of essential amino acids or cytotoxic molecules that could be selectively transported by SLC6A14) are also being actively explored for other tumor types which have been shown to overexpress SLC6A14 [18, 42].

## 5. Conclusion

SLC6A14 appears to be a good candidate transporter for further exploration as a functional imaging target for PDAC that could complement existing anatomical imaging techniques for both diagnosis and staging.

## Figures and Tables

**Table 1:** Gene normalized levels of SLC6A14 mRNA.

Patient #	Adjacent normal duct	Adjacent normal acinar	Distant normal acinar	Tumor	Tumor/normal^1^
1	2.54			138.68	54.60
2	0.84			36.16	43.05
3	0.17			1.51	8.88
4	1.16			119.89	103.35
5	10.31	0.13		62.44	6.06
6	1.34	0.10		11.82	8.82
7	0.29			7.83	27.00
8	1.10			29.73	27.03
9	6.66	0.37		54.33	8.16
10	0.22			342.69	1557.68
11	0.38	0.13		22.01	57.92
12	2.60			27.22	10.47
13	2.26			11.24	4.97
14	8.94			102.6	11.48
15	0.36			34.56	96.00
16	4.44	0.03		10.00	2.25
17 (CT-iso)			0.03	13.10	436.67
18 (CT-iso)			0.03	22.56	752.00
19 (CT-iso)			0.10	103.03	1030.30
20 (CT-iso)			0.02	439.30	21965.00
21 (CT-iso)			0.04	15.49	387.25

CT-iso, computed tomography-isoattenuating.

^1^Adjacent normal duct was used for the calculation in patients 1–16. Distant normal acinar was used for patients 17–21. Values are in RPKM (mapped reads per kilobase per million mapped reads).

**Table 2:** SLC6A14 IHC staining intensity.

	0: none (*N* = 5)	1: weak (*N* = 50)	2: moderate (*N* = 28)	3: strong (*N* = 11)	Total (*N* = 94)	*p* value
Survival						0.1181
*N*	5	42	24	8	79
Events	5	36	20	7	68
Median survival days	633.0 (487.0–2532.0)	733.0 (405.0–1247.0)	595.0 (334.0–1281.0)	366.0 (253.0–641.0)	579.0 (419.0–962.0)
2 Yr survival rate	40.0% (0.0%–82.9%)	51.2% (35.9%–66.5%)	45.8% (25.9%–65.8%)	0.0%	44.2% (33.1%–55.3%)
Year 2 *N* at risk	2	21	11	0	34
Age of onset						0.1099
*N*	5	37	22	8	72
Mean (SD)	73.0 (7.0)	67.6 (10.6)	66.5 (9.2)	61.1 (9.0)	66.9 (10.0)
Median	75.0	69.0	70.0	59.5	69.0
Q1, Q3	74.0, 76.0	61.0, 75.0	60.0, 75.0	54.0, 68.0	60.0, 75.0
Range	(61.0–79.0)	(37.0–85.0)	(48.0–76.0)	(50.0–76.0)	(37.0–85.0)
Median age of onset	75.0 (61.0–79.0)	69.0 (64.0–73.0)	70.0 (60.0–74.0)	59.5 (50.0–69.0)	69.0 (66.0–72.0)	0.0978
Sex						0.2142
Missing	0	8	4	3	15
Female	1 (20.0%)	25 (59.5%)	10 (41.7%)	3 (37.5%)	39 (49.4%)
Male	4 (80.0%)	17 (40.5%)	14 (58.3%)	5 (62.5%)	40 (50.6%)
Obesity						0.2358
Missing	1	21	9	5	36
BMI < 30	2 (50.0%)	24 (82.8%)	17 (89.5%)	4 (66.7%)	47 (81.0%)
BMI 30+	2 (50.0%)	5 (17.2%)	2 (10.5%)	2 (33.3%)	11 (19.0%)
Diabetes self-reported						0.6592
Missing	0	8	4	3	15
No DM	5 (100.0%)	33 (78.6%)	18 (75.0%)	6 (75.0%)	62 (78.5%)
DM	0 (0.0%)	9 (21.4%)	6 (25.0%)	2 (25.0%)	17 (21.5%)
Pancreatitis self-reported						0.8622
Missing	0	8	4	3	15
No pancreatitis	4 (80.0%)	31 (73.8%)	18 (75.0%)	7 (87.5%)	60 (75.9%)
Pancreatitis	1 (20.0%)	11 (26.2%)	6 (25.0%)	1 (12.5%)	19 (24.1%)
Tumor grade						0.5917
2	0 (0.0%)	8 (16.0%)	1 (3.6%)	1 (9.1%)	10 (10.6%)
3	4 (80.0%)	31 (62.0%)	20 (71.4%)	6 (54.5%)	61 (64.9%)
4	1 (20.0%)	11 (22.0%)	7 (25.0%)	4 (36.4%)	23 (24.5%)

## Abbreviations

cDNA: Complementary DNA
CT: Computed tomography
FFPE: Formalin-fixed paraffin-embedded
^18^FDG: 2-Deoxy-2-[^18^F]fluoro-D-glucose
^18^FDG-PET: 2-Deoxy-2-[^18^F]fluoro-D-glucose positron emission tomography
LCM: Laser capture microdissection
MAP-RSeq: Mayo Analysis Pipeline for RNA Sequencing
MRI: Magnetic resonance imaging
NIS: Sodium iodide symporter
PDAC: Pancreatic ductal adenocarcinoma
PET: Positron emission tomography
RNase H: Ribonuclease H
RNASeq: RNA sequencing
SLC: Solute carrier
SPECT: Single-photon emission computed tomography
SPIA: Single Primer Isothermal Amplification
SPORE: Specialized Program of Research Excellence
TMA: Tissue microarray.
